# Apple as a source of dietary phytonutrients: an update on the potential health benefits of apple

**DOI:** 10.17179/excli2016-483

**Published:** 2016-09-19

**Authors:** Tae Kyung Hyun, Keum-Il Jang

**Affiliations:** 1 Department of Industrial Plant Science and Technology, College of Agricultural, Life and Environmental Sciences, Chungbuk National University, Cheongju 361-763, Republic of Korea; 2 Department of Food Science and Biotechnology, College of Agricultural, Life and Environmental Sciences, Chungbuk National University, Cheongju 361-763, Republic of Korea

## ⁯

Dear Editor,

Since several studies have demonstrated the pharmacological activities (anti-oxidant, anti-microbial, anti-inflammatory, anti-diabetic, anti-cancer, etc.) of fruits and vegetables, it has been suggested that a daily intake of apples is associated with the prevention of several chronic diseases, including chronic obstructive pulmonary disease, asthma and different types of cancers (Boeing et al., 2012[1]; Kalinowska et al., 2014[15]). 

Apples, the world's second most consumed fruit after bananas, contain several nutrients together with non-nutrients such as dietary fiber, minerals and vitamins. In addition, apples possess rich contents of polyphenols, which are divided into several groups including hydroxybenzoic acids, hydroxycinnamic acids and their derivatives, flavonols, dihydrochalcones, anthocyanids, monomeric flavanols and oligomeric flavanols (Kalinowska et al., 2014[15]). Due to the high nutraceutical values and various polyphenols of apples, apples have exhibited beneficial effects on the health against cancer, asthma and pulmonary dysfunction, cardiovascular diseases, Alzheimer's disease, decline of normal aging, weight management and diabetes (Hyson, 2011[11]). These findings have supported the age-old saying “an apple a day keeps the doctor away”. 

The present report summarizes key recent studies that have demonstrated the biological and pharmacological properties of apple and its products (Table 1(Tab. 1)) (References in Table 1: Anti-cancer: Delphi et al., 2015[5]; Walia et al., 2014[28]; Schiavano et al., 2015[25]; Hung et al., 2015[10]; Li et al., 2014[21]; Kao et al., 2015[16]; Qiao et al., 2015[23]; Jedrychowski et al., 2010[12]; Le Marchand et al., 2000[19]; Anti-obesity and anti-diabetic effects: Jiang et al., 2016[14]; Sun et al., 2016[26]; Sampath et al., 2016[24]; O'Neil et al., 2015[22]; Bouderbala et al., 2016[2]; Fathy and Drees, 2016[7]; Dange and Deshpande, 2013[4]; Knekt et al., 2002[17]; Anti-inflammation: Jensen et al., 2014[13]; Espley et al., 2014[6]; Lee et al., 2014[20]; Hepato-protective: Cheng et al., 2014[3]; Krajka-Kuźniak et al., 2015[18]; Antigenotoxicity: Gomes de Moura et al., 2015[8]; Reduction of cardiotoxicity: Vineetha et al., 2014[27]; Etc.: Hodgson et al., 2016[9]). We hope that this report will further spur the research on the potential application of apple, its products and its biologically active compounds for preventing several chronic diseases in humans.

## Acknowledgements

This study was supported by Chungbuk Industrial Academic Institutional Consortium for Apple.

## Conflict of interest

The authors declare no conflict of interest.

## Figures and Tables

**Table 1:**
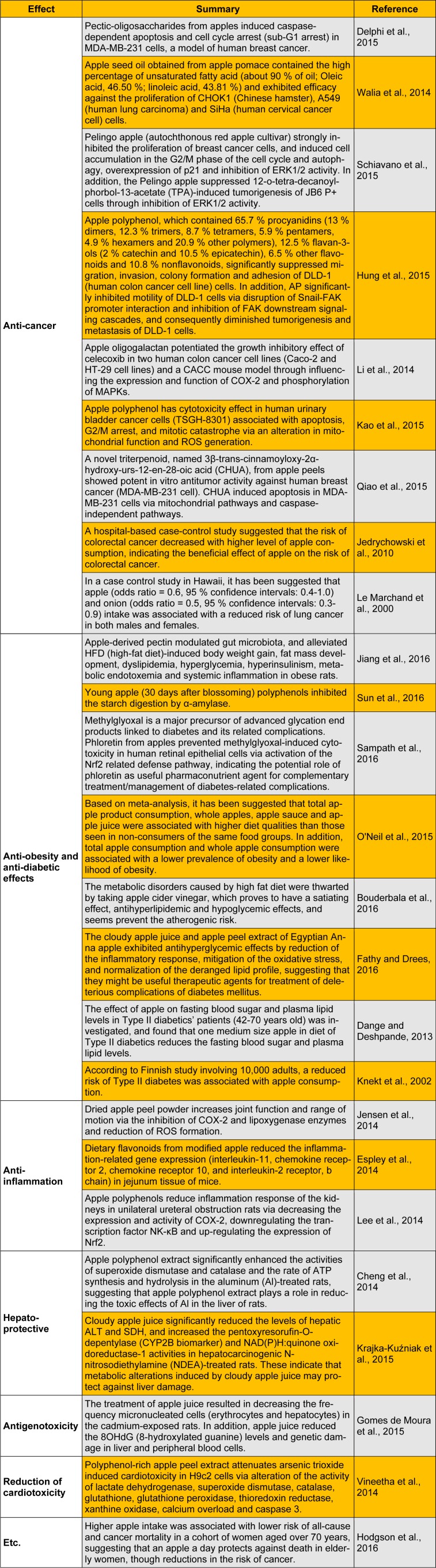
Recent studies on biological and pharmacological activities of apple and its products
